# Unresolved Splenomegaly in Recently Resettled Congolese Refugees ― Multiple States, 2015–2018

**DOI:** 10.15585/mmwr.mm6749a2

**Published:** 2018-12-14

**Authors:** Laura D. Zambrano, Olivia Samson, Christina Phares, Emily Jentes, Michelle Weinberg, Matthew Goers, S. Patrick Kachur, Robert McDonald, Bozena Morawski, Henry Njuguna, Yasser Bakhsh, Rebecca Laws, Corey Peak, Sally Ann Iverson, Carla Bezold, Hayder Allkhenfr, Roberta Horth, Jun Yang, Susan Miller, Michael Kacka, Abby Davids, Margaret Mortimer, Nomana Khan, William Stauffer, Nina Marano

**Affiliations:** ^1^Epidemic Intelligence Service, CDC; ^2^Division of Global Migration and Quarantine, National Center for Emerging and Zoonotic Infectious Diseases, CDC; ^3^Oak Ridge Institute for Science and Education, Oak Ridge, Tennessee; ^4^Division of Global Health Protection, Center for Global Health, CDC; ^5^Division of Parasitic Diseases and Malaria, Center for Global Health, CDC; ^6^New York State Department of Health; ^7^Idaho Department of Health and Welfare; ^8^Washington State Department of Health, Tumwater, Washington; ^9^California Department of Public Health, Richmond, California; ^10^Arizona Department of Health Services; ^11^Utah Department of Health; ^12^Pennsylvania Department of Human Services; ^13^South Carolina Department of Health and Environmental Control; ^14^Family Medicine Residency of Idaho, Boise, Idaho; ^15^University of Minnesota, Minneapolis.

## Abstract

In 2014, panel physicians from the International Organization for Migration (IOM), who conduct Department of State–required predeparture examinations for U.S.-bound refugees at resettlement sites in Uganda, noticed an unusually high number of Congolese refugees with enlarged spleens, or splenomegaly. Many conditions can cause splenomegaly, such as various infections, liver disease, and cancer. Splenomegaly can result in hematologic disturbances and abdominal pain and can increase the risk for splenic rupture from blunt trauma, resulting in life-threatening internal bleeding. On CDC’s advice, panel physicians implemented an enhanced surveillance and treatment protocol that included screening for malaria (through thick and thin smears and rapid diagnostic testing), schistosomiasis, and several other conditions; treatment of any condition identified as potentially associated with splenomegaly; and empiric treatment for the most likely etiologies, including malaria and schistosomiasis. CDC recommended further treatment for malaria with primaquine after arrival, after glucose-6-phosphate dehydrogenase testing, to target liver-stage parasites. Despite this recommended treatment protocol, 35 of 64 patients with available follow-up records had splenomegaly that persisted beyond 6 months after resettlement. Among 85 patients who were diagnosed with splenomegaly through abdominal palpation or ultrasound at any point after resettlement, 53 had some hematologic abnormality (leukopenia, anemia, or thrombocytopenia), 16 had evidence of current or recent malaria infection, and eight had evidence of schistosomiasis. Even though primaquine was provided to a minority of patients in this cohort, it should be provided to all eligible patients with persistent splenomegaly, and repeated antischistosomal therapy should be provided to patients with evidence of current or recent schistosomiasis. Given substantial evidence of familial clustering of cases, family members of patients with known splenomegaly should be proactively screened for this condition.

Approximately 6 months before resettlement, all United States–bound refugees undergo a medical examination overseas conducted by panel physicians appointed by the U.S. Department of State, in accordance with technical instructions provided by CDC (*1*). In March and July 2015, among 987 refugees undergoing overseas medical examination in Uganda, 145 (14.7%) bound for 23 U.S. states^†^ had palpable but presumably asymptomatic splenomegaly, prompting further investigation. This initial investigation failed to identify a clear etiology, but malaria was considered to be one of the potential causes (*2*). Because of the uncertain etiology, CDC established a mechanism for domestic U.S. clinicians to report postarrival clinical outcomes and receive guidance in the event that case management guidelines changed. The literature published on malaria-associated splenomegaly indicates that the condition usually resolves within months of departure from an area of malaria endemicity (*3*,*4*). However, throughout 2016, it became evident that despite implementation of the diagnostic and treatment protocol,^§^ splenomegaly was not resolving in some patients. In response, on April 25, 2017, IOM asked CDC to investigate unresolved splenomegaly (defined as any palpable splenomegaly after arrival) among refugees with a diagnosis of splenomegaly before or after arrival to inform overseas and postarrival screening exams and clinical management. Goals were to describe associated or underlying conditions, clinician management strategies, and clinical outcomes.

CDC contacted the 10 states with the highest number of patients with splenomegaly, as well as Georgia, because of its geographic proximity to CDC. Among these 11 states, nine^¶^ agreed to participate. Investigators obtained data through retrospective medical chart abstractions from all available postarrival clinical records and asked participating health care providers about additional cases of splenomegaly among all arriving Congolese refugees. Investigators also collected laboratory results suggestive of potential etiologies, such as total immunoglobulin M for differential diagnosis of tropical splenomegaly (*5*); available malaria testing results (by smear microscopy, rapid diagnostic testing, or molecular testing); stool specimen testing; urinalyses; and serological evidence of prior schistosomiasis with current eosinophilia (*6*); and then recorded clinical progress and hematologic and hepatic outcomes.

Overall, 135 Congolese refugees with splenomegaly who resettled within the nine states during April 2015–May 2017 were identified; 90 (66.6%) patients were clustered in 22 families. Postarrival medical records were available for 117 (87%) patients, including 96 who received a diagnosis overseas (86 from the original cohort identified prospectively by IOM and an additional 10 patients who were identified by retrospective review of medical records by domestic clinicians) and 21 patients who received a diagnosis domestically (Table 1) (Figure).** Clinicians in New York identified six cases by proactively screening family members of patients with known splenomegaly. All initial domestic screening examinations occurred within 90 days of arrival, as recommended by CDC (*7*). At this postarrival examination, splenomegaly was noted for 64 (66.7%) of 96 patients who received a diagnosis overseas and 21 patients with a domestic diagnosis, resulting in a total of 85 patients who had splenomegaly after their arrival.

**TABLE 1:** States of resettlement of Congolese refugees* with splenomegaly ― United States, 2015–2018

State	Diagnosed overseas		Diagnosed domestically and identified after arrival	Total no. included in investigation^†^	No. with splenomegaly at initial exam
	Member of original cohort (identified prospectively)	Identified retrospectively after arrival			
Arizona	12	2	0	14	5
California	12	0	1	13	9
Georgia	1	2	0	3	3
Idaho^§^	13	1	0	14	11
New York	12	1	9	22	18
Pennsylvania	11	1	0	12	4
South Carolina	6	0	7	13	11
Utah	13	3	2	18	18
Washington	6	0	2	8	6
**Total**	**86**	**10**	**21**	**117**	**85**

* Patients with splenomegaly in a cohort investigated overseas (https://www.cdc.gov/mmwr/volumes/65/wr/mm6535a5.htm?s_cid=mm6535a5) and cases identified after arrival across the eight most affected states and Georgia are shown, along with the total number of patients included in this investigation and number of patients with splenomegaly identified at the initial exam.

^†^ Eighteen additional records from Idaho are pending.

^§^ Data collection is ongoing in Idaho; case records have been submitted for 14 of 32 known cases in the state.

**FIGURE:**
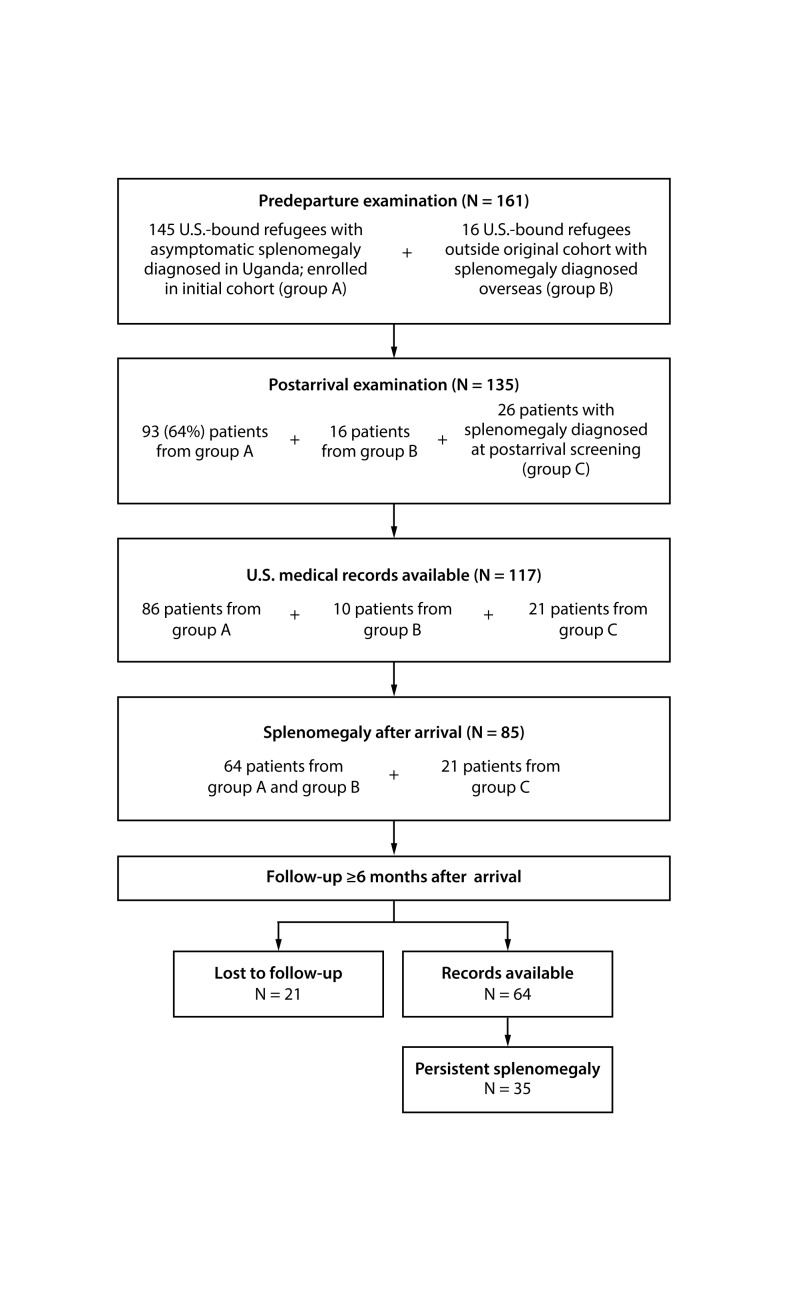
Number of Congolese refugees with unresolved splenomegaly, by stage of resettlement — United States, 2015–2018*^,†,§^ * Among refugees receiving predeparture examinations (N = 161), 145 resettling to 23 states were enrolled in the initial cohort (Group A). https://www.cdc.gov/mmwr/volumes/65/wr/mm6535a5.htm?s_cid=mm6535a5. ^†^ Among refugees receiving postarrival examinations (N = 135), 93 resettled in nine participating states: Arizona, California, Georgia, Idaho, New York, Pennsylvania, South Carolina, Utah, and Washington (group A). ^§^ Group C patients were screened in six states: California, Idaho, New York, South Carolina, Utah, and Washington.

Among all 85 patients with splenomegaly at their initial examination, 64 (75.3%) had at least one clinic visit (for any condition) >6 months after arrival. Among these 64 patients, median duration between arrival and the last visit when splenomegaly was noted was 9.0 months (range = 0.3–27.9 months), and 35 (54.7%) had persistent splenomegaly, defined as a palpable spleen 6 months after departing an area with endemic malaria.

## Hematologic, Hepatic, and Infectious Disease Screening

Predeparture or postarrival laboratory results were available for 84 of 85 refugees with documented splenomegaly and 24 of 32 without documented splenomegaly at their initial domestic exam (Table 2). Among the 84 with splenomegaly, 53 (63.1%) had a hematologic abnormality, such as anemia (43 of 83, 51.8%), leukopenia (16 of 79, 20.3%), or thrombocytopenia (19 of 34, 55.9%). Elevated liver enzymes, including alanine transaminase or aspartate transaminase, were present in 11 (20.0%) of 55 patients, and elevated alkaline phosphatase was present in 31 (53.5%) of 58 patients with available results. Among the 46 patients who were screened for malaria by thin or thick smear after arrival, six (13.0%) were smear-positive; two of these six patients had evidence of infection with *Plasmodium falciparum*, four had evidence of infection with *Plasmodium vivax* or *Plasmodium ovale*, and two had coinfection with *Plasmodium malariae* and *P. vivax* or *P. ovale*. Among 30 refugees with splenomegaly after arrival for whom *Schistosoma* immunoglobulin G results were available, 15 (50.0%) had evidence of prior infection; among 13 with both *Schistosoma* immunoglobulin G results and an eosinophil count, eight (61.5%) had eosinophilia (Table 2).

**TABLE 2:** Underlying conditions and clinical sequelae in Congolese refugees with splenomegaly diagnosed predeparture or post-arrival in the United States, by presence of splenomegaly at the initial domestic exam after arrival — nine states,* 2015–2018

Laboratory test results	Reference range^†^	Initial domestic exam			
		Splenomegaly (n = 85)^§^		No splenomegaly (n = 32)^¶^	
		No. tested	No. with condition (%)	No. tested	No. with condition (%)
Elevated total IgM**	**46–304 mg/dL**	**27**	**12 (44.4)**	**0**	**0**
**Malaria (smear or RDT-positive)**	N/A	55	16 (29.1)	19	10 (52.6)
**Elevated *Schistosoma* IgG**	≥0.20 OD	30	15 (50.0)	4	1 (25.0)
**Eosinophilia**	≥500 cells/*μ*L	77	21 (27.3)	21	5 (23.8)
Among *Schistosoma* IgG(+)	—	13	8 (61.5)	1	0 (0)
Among *Schistosoma* IgG(-)	—	15	2 (13.3)	2	1 (50.0)
**Other hematologic abnormality**	N/A	84	53 (63.1)	24	11 (45.8)
**Leukopenia**	<4,000 cells/*μ*L	79	16 (20.3)	24	3 (12.5)
**Anemia (hemoglobin)**	F: ≤12.0 g/dL; M: ≤14.0 g/dL	83	43 (51.8)	24	10 (41.7)
**Thrombocytopenia**	<150,000 platelets/*μ*L	34	19 (55.9)	8	3 (37.5)
**Elevated alkaline phosphatase**	>147 IU/L	58	31 (53.5)	12	8 (66.7)
**Elevated transaminases**	>40 IU/L	55	11 (20.0)	11	1 (9.1)
Elevated AST	—	55	9 (16.4)	11	1 (9.1)
Elevated ALT	—	55	8 (14.6)	11	1 (9.1)

**Abbreviations:** ALT = alanine aminotransferase; AST = aspartate aminotransferase; F = females; IgG = immunoglobulin G; IgM = immunoglobulin M; M = males; OD = optical density; RDT = rapid diagnostic test.

* Arizona, California, Idaho, New York, Pennsylvania, South Carolina, Utah, Washington, and Georgia.

^†^ In the absence of laboratory reference ranges from all laboratories the highest cutoff value reported was used, which might have sacrificed sensitivity.

^§^ Includes 63 patients with a diagnosis of splenomegaly overseas who had splenomegaly noted at their initial domestic exam and 21 patients who received a diagnosis of splenomegaly domestically. Eighty-five patients had splenomegaly on arrival, but laboratory records were absent for one.

^¶^ Includes 24 patients with available laboratory results who received a diagnosis of splenomegaly overseas, none of whom had splenomegaly noted at their initial domestic exam, excluding eight patients for whom laboratory results were not available.

** Total IgM is used for in the diagnosis of hyperreactive malarial splenomegaly syndrome.

## Treatment

All patients were treated empirically with praziquantel for schistosomiasis and at least 1 dose of artemether-lumefantrine for malaria before departure. Although CDC recommended treatment with primaquine after arrival for all Congolese refugees with splenomegaly (*2*), only 31 (26.5%) of 117 patients had documentation of primaquine administration in their postarrival medical charts, and none had documentation of completion of the 14-day regimen. Among the 31 patients who received primaquine, 29 (93.6%) had a clinic visit >6 months after arrival, compared with 43 (50.0%) of 86 patients who did not receive primaquine. Among these 29 patients, the median duration of observed splenomegaly was 12.4 months after arrival (range = 0.3–24.1 months), and 20 (69.0%) met the definition for persistent splenomegaly. Three patients received praziquantel after arrival.

## Discussion

Few data, beyond anecdotal clinician reports, exist on tropical splenomegaly, and patients’ anticipated clinical course is still largely unknown, particularly after relocation to nontropical environments. In contrast to what has been reported previously (*3*,*4*), many of the patients in this report had persistent splenomegaly long after arrival, despite receipt of a short course of malaria treatment and removal from an area with endemic malaria, indicating that the clinical course of tropical splenomegaly is still poorly understood. Malaria might still be the predominant underlying etiology, particularly given the presence of species including *P. vivax* and *P. ovale*, which can cause relapsing disease, in some refugees. The original recommendation (*2*) remains unchanged: all refugees of Congolese origin with splenomegaly should receive presumptive treatment with primaquine after arrival in the United States. Despite this recommendation, two thirds of refugees identified with splenomegaly in this investigation did not receive primaquine. Lack of awareness among domestic physicians, need for repeated visits for glucose-6-phosphate dehydrogenase testing, a long (14-day) course, safety concerns, and availability of primaquine might have contributed to inconsistent administration.

The majority of patients with persistent splenomegaly had some combination of hematologic abnormalities, potentially caused by splenic sequestration. Many patients also had elevated liver transaminases, suggesting a need to monitor hepatic complications in this population. In light of the high proportion of patients with evidence of prior *Schistosoma* infection (47%) or eosinophilia (22%), it is important for physicians to consider further screening and diagnostic evaluation through stool and urine examination for ova or urinalysis for red blood cells. Among patients with persistent splenomegaly and clinical indicators of *Schistosoma* infection, such as eosinophilia without any other known cause, clinicians should consider repeating antischistosomal therapy with praziquantel. In addition, because etiology might be multifactorial or patient-specific, clinicians also need to consider further diagnostic testing in cases of persistent splenomegaly for Epstein-Barr virus, autoimmune disorders, or oncologic/hematologic etiologies.

The findings in this report are subject to at least four limitations. First, because data were obtained from clinic visits that occurred at irregular intervals, these findings likely underestimate the duration of splenomegaly in this population. Second, because of loss to follow-up, this investigation cannot estimate the actual proportion of patients whose condition resolved after their initial screening exam. Third, data quality varied widely across clinics, and diagnostic information from U.S.-based clinics (particularly more sensitive molecular diagnostics) was unavailable in most instances. Finally, considering the 21 cases of splenomegaly identified after U.S. arrival, the condition was likely underdiagnosed. Increased awareness and emphasis on careful spleen examination might improve sensitivity of predeparture detection.

Despite published reports suggesting that resolution would follow malaria treatment and removal from an area with endemic malaria (*3*,*4*), this analysis found that splenomegaly persisted after arrival in many Congolese refugees, in some cases beyond 2 years. Associated pathologic conditions, such as anemia and thrombocytopenia, also were prevalent. Clinicians caring for such patients both predeparture and postarrival need to be aware of the high prevalence of splenomegaly in this population. Given familial clustering and additional cases identified through proactive family screening, both overseas and domestic clinicians could consider screening family members of Congolese refugees with splenomegaly. Congolese refugees found to have splenomegaly should be treated with primaquine, if eligible; counseled on the condition and precautions (e.g., avoidance of contact sports); followed closely; and referred for specialty care if they fail to respond to treatment. Multiple etiologies are possible, but there is likely a predominant underlying infection and immune response. Future investigations might further reveal associated pathologies and etiologies of tropical splenomegaly in this population.

**Summary**

**What is already known about this topic?**

Since 2014, a large number of resettling Congolese refugees have been found to have splenomegaly, which has not resolved in some patients despite treatment.

**What is added by this report?**

Despite recommendations, most refugees with splenomegaly did not have documented receipt of primaquine after resettlement. Most patients were clustered within families. Approximately 50% of patients with available medical records had persistent splenomegaly >6 months after arrival; 63% of patients with splenomegaly had a hematologic abnormality.

**What are the implications for public health practice?**

Eligible Congolese refugees with splenomegaly should be treated with primaquine, followed closely, and referred for specialty care if they fail to respond to treatment, and their family members should be proactively screened for splenomegaly.

## Footnotes

* These authors contributed equally.

† The 23 states were Arizona, California, Connecticut, Florida, Georgia, Idaho, Illinois, Indiana, Kansas, Kentucky, Massachusetts, Michigan, Nevada, New Hampshire, New York, Ohio, Pennsylvania, South Carolina, South Dakota, Texas, Utah, Vermont, and Washington.

¶ Eight of the 10 states with the highest number of patients with splenomegaly (Arizona, California, Idaho, New York, Pennsylvania, South Carolina, Utah, and Washington) and Georgia agreed to participate.

** Among the 31 patients classified as having splenomegaly after arrival, 23 originated in Uganda and eight in Tanzania. Data are pending for an additional 11 patients with splenomegaly who arrived from Uganda, Tanzania, and Burundi.
